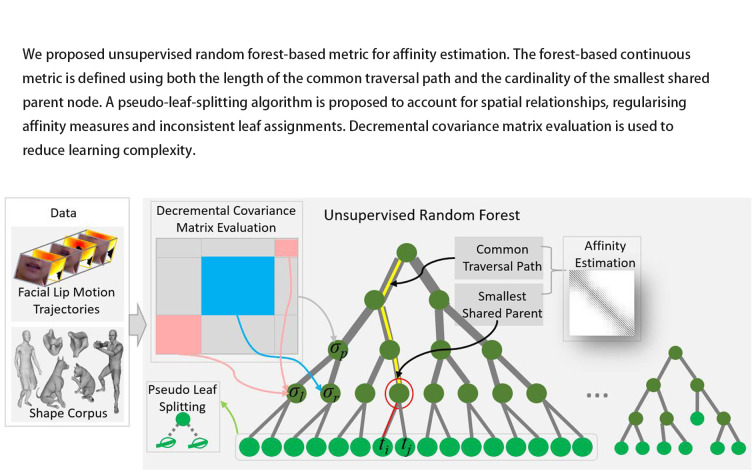# Unsupervised random forest for affinity estimation

**DOI:** 10.1007/s41095-021-0241-9

**Published:** 2021-12-06

**Authors:** Yunai Yi, Diya Sun, Peixin Li, Tae-Kyun Kim, Tianmin Xu, Yuru Pei

**Affiliations:** 1 Key Laboratory of Machine Perception (MOE), Department of Machine Intelligence, Peking University, Beijing, 100871 China; 2 Department of Electrical and Electronic Engineering, Imperial College London, London, UK; 3 School of Stomatology, Stomatology Hospital, Peking University, Beijing, 100081 China

**Keywords:** affinity estimation, forest-based metric, unsupervised clustering forest, pseudo-leaf-splitting (PLS)

## Abstract

This paper presents an unsupervised clustering random-forest-based metric for affinity estimation in large and high-dimensional data. The criterion used for node splitting during forest construction can handle rank-deficiency when measuring cluster compactness. The binary forest-based metric is extended to continuous metrics by exploiting both the common traversal path and the smallest shared parent node.

The proposed forest-based metric efficiently estimates affinity by passing down data pairs in the forest using a limited number of decision trees. A pseudo-leaf-splitting (PLS) algorithm is introduced to account for spatial relationships, which regularizes affinity measures and overcomes inconsistent leaf assign-ments. The random-forest-based metric with PLS facilitates the establishment of consistent and point-wise correspondences. The proposed method has been applied to automatic phrase recognition using color and depth videos and point-wise correspondence. Extensive experiments demonstrate the effectiveness of the proposed method in affinity estimation in a comparison with the state-of-the-art.

## 1 Introduction

Affinity estimation is an essential step in various computer vision and image processing tasks. Affinity of motion trajectories, for example, is utilized in motion segmentation [1, 2] and action recognition [3]. Automatic phrase recognition employs trajectory affinity to define motion patterns in color and depth videos [4]. Point-to-point affinity and shape correspondence are essential for attribute transfer and data reuse [5–9], as well as shape comparisons in morphological studies [10, 11]. It is, however, time consuming to estimate pairwise affinities for large-scale datasets, where the complexity grows quadratically with the size of the dataset. Some distance metrics, such as the earth mover's distance, have higher computational costs for higher-dimensional data. This paper presents an unsupervised random-forest-based metric for efficient affinity estimation, and demonstrates its efficacy on automatic phrase recognition and point-wise correspondence of a shape corpus.

Random forests have been popular in computer vision for decades, and are well-known for their scalability and real-time evaluation as well as providing good generalization to unseen data [12–18]. A clustering random forest works in an unsupervised fashion [19–25] to estimate the underlying data distribution and affinity without prior labels. Alzubaidi et al. [26] utilized a density forest [20] with a Gaussian distribution assumption for tree nodes, where clustering compactness was measured by the covariance matrix. However, the zero-valued determinant in the case of rank-deficiency causes the criterion to become invalid. The combinatorial node splitting criterion which integrates trace-based distribution measurement and scatter index [4] can handle rank-deficiency for optimal node splitting.

Recent research addressed forest-based metrics for affinity estimation. The cascaded clustering forest (CGF) was proposed to refine voxel-wise affinity

1 Key Laboratory of Machine Perception (MOE), Department of Machine Intelligence, Peking University, Beijing 100871, China. E-mail: Y. Yi, yiyunai521@126.com; D. Sun, dysun@pku.edu.cn; P. Li, lipeixin@pku.edu.cn; Y. Pei, yrpei@pku.edu.cn (✉).

2 Department of Electrical and Electronic Engineering, Imperial College London, London, UK. E-mail: tk.kim@imperial.ac.uk.

3 School of Stomatology, Stomatology Hospital, Peking University, Beijing 100081, China. E-mail: tmxuortho@163.com.

Manuscript received: 2021-03-29; accepted: 2021-05-26

by iteratively updating geodesic coordinates [27] using a set of clustering models. The mixed metric random forest (MMRF) utilized self-learning of data distributions for matching consistencies between images [28], taking advantage of the weak labeling and classification criterion to optimize node splitting. The oblique clustering forest (OCF) [29] extended the splitting criterion from traditional orthogonal hyperplanes to oblique hyperplanes, reducing the tree depth and model complexity. The spatially consistent (SC) clustering forest employed a data-dependent learning guarantee for unsupervised clustering of randomized trees [30]. The above clustering forests introduce additional computation, such as cascaded clustering models [27], fine-tuning with penalized weighting of the classification entropy [28], dominant principal component and regression [29], and data-dependent learning guarantee for tree pruning [30], to improve data clustering and affinity estimation. In contrast, our work here does not introduce additional computational costs to construct the clustering forest. Instead, we extend the binary forest-based metric to a continuous one for affinity estimation. As training an unsupervised clustering forest is typically more time-consuming than a supervised classification forest due to entropy estimation for the high-dimensional data, the decremental covariance matrix evaluation technique is introduced to avoid assessment of covariance matrices from scratch and reduce the learning complexity.

Affinities are measured efficiently by hierarchical clustering forests, in contrast to the learning-based feature fusion for the affinity graph using iterative optimization of convex problems [31]. Two points are intuitively assumed to be similar if they are placed in the same leaf. The generalized forest-based metric is derived from the average affinities of individual trees; it has been used to measure data similarity [20, 24]. A continuous affinity measure has been proposed based on the common traversal path from the root to leaf nodes as well as the node cardinality on the path [25]. To relieve the weight computation on the traversal path, we present a forest-based metric as a linear combination of normalized common-traversal-path-based and smallest-shared-parent-based metrics. The proposed metric takes into account both the unbalanced data distribution and partial similarity. Given the pairwise affinities of a dataset, it is straightforward to compute the low-dimensional embedding. Ganapathi-Subramanian et al. [32] constructed a joint latent embedding function combining diffusion embedding and a linear mapping for descriptor transport in a shape corpus, where the nonlinear embedding function relied on the predefined feature descriptors. The paper addresses the forest-based metric and affinity estimation. The embedding is conducted by the multi-dimensional scaling (MDS) algorithm [33]; it is computed based on affinity estimation without explicit representation learning.

This work introduces a pseudo-leaf-splitting (PLS) algorithm to handle inconsistent leaf assignments, since the random forest built upon independent data points cannot accommodate global data structures. The random-forest-based metric with PLS regularizes point-wise correspondences. The proposed PLS technique differs from existing methods [9, 34–36] in that it bridges the gap between separate point-wise correspondence and consistency refinements. Deep learning-based methods have been used for shape correspondence [5, 37–39], learning from prior ground truth correspondences or metric space alignment. 3DN [39] and 3D-coded [38] were unsupervised end-to-end networks to infer global displacement fields between a shape and a template, utilizing chamfer and earth mover's distance-based loss functions. FMNet [37] optimized a feature extraction network via a low-dimensional spectral map. ADD3 used anisotropic diffusion-based spectral feature descriptors [5]. FMNet [37] and ADD3 [5] learn in a supervised manner, requiring prior ground truth correspondence. Unlike deep neural network-based descriptor learning, this work exploits unsupervised forest-based metric learning for point-wise correspondence.

This paper presents a combined forest-based metric and a PLS regularization scheme to improve the forest-based metric for affinity estimation, as shown in Fig. 1. The main contributions of this work are: (i) a continuous forest-based metric exploiting both the common traversal path and the cardinality of the smallest shared parent node, enabling efficient and effective affinity estimation in large and high-dimensional data, (ii) a PLS scheme to regularize the forest-based metric to account for global spatial and structural relationships, overcoming inconsistent leaf

**Fig. 1** Our proposed unsupervised random forest-based metric for affinity estimation. The forest-based continuous metric is defined using both the length of the common traversal path and the cardinality of the smallest shared parent node. A pseudo-leaf-splitting algorithm is proposed to account for spatial relationships, regularising affinity measures and inconsistent leaf assignments. Decremental covariance matrix evaluation is used to reduce learning complexity.

assignments, and (iii) experimental demonstrations and comparisons with the state-of-the-art indicating successful affinity estimation for facial trajectories and 3D points, enabling efficient and automatic phrase recognition and consistent correspondences for a 3D shape corpus.

## 2 Unsupervised random forest

Given an unlabeled dataset $T = \{t_i | i = 1, \dots, N\}$, comprising a set of trees trained independently, the unsupervised density forest estimates the underlying data distribution using a Gaussian distribution assumption [20]. The combinatorial node splitting criterion integrates a trace-based distribution measurement and a scatter index [4]. The objective function $I$ of the $j$-th node with data $T_j$ is defined as follows.

$$I = -\sum_{i=l,r} \frac{m_{T_j^i}}{m_{T_j}} \ln \left( \mathrm{tr} \left( \sigma(T_j^i) \right) \right) + \lambda \frac{\|\mu_l - \mu_r\|_\infty}{\sum_{i=l,r} \phi(T_j^i, \mu_i)} \quad (1)$$

where $\mathrm{tr}(\cdot)$ is the matrix trace, $T_j^i$ denotes the data assigned to the $i$-th child node from parent node $j$, $\sigma$ denotes the covariance matrix of the Gaussian distribution, $m_{T_j^i}$ denotes the size of the left or the right child nodes, and $m_{T_j}$ the parent node size. $\phi(T_j^i, \mu_i) = \max_{t \in T_j^i} \|t - \mu_i\|_\infty$. $\mu_l$ and $\mu_r$ are the centroids of the left and right child nodes respectively. The constant $\lambda$ is set to 50 empirically.

The covariance matrices need to be repeatedly evaluated when given randomly selected parameters; it is time-consuming to evaluate the covariance matrix $\sigma$ from scratch for the optimal splitting parameters when building the forest. This work introduces a decremental covariance matrix evaluation technique (see Appendix A). The complexity of covariance matrix evaluation is reduced from $O(m\rho^2)$ to $O(\rho)$ by the decremental technique, where $m$ is the cardinality of the node, and $\rho$ denotes the data dimensionality. The trace evaluation complexity is reduced to $O(\kappa\rho)$ given $\kappa$ randomly selected parameters.

## 3 Forest-based affinity estimation

### 3.1 Binary forest-based metric

The forest leaves $L$ define a partition of the training data. When feeding an instance $t$ to a tree, it will finally reach a leaf $\ell(t) \in L$, after a sequence of binary tests stored in the branch nodes. Instances assigned to the same leaf node are assumed to be similar and their pairwise affinity is set to 1; it is 0 otherwise. The symmetric affinity matrix $\mathcal{A}$ is defined as a weighted combination of $\mathcal{A}_k$ from independent trees.

$$\mathcal{A} = \frac{1}{n_T} \sum_{k=1}^{n_T} \mathcal{A}_k \quad (2)$$

where $n_T$ is the number of trees. Since only points within a leaf node are considered to be similar, the affinity matrix from the random forest automatically accounts for neighboring relationships. Thus, $\mathcal{A}$ can be viewed as a geodesic affinity matrix of the original dataset. However, when using the $L_2$ distance metric, there is no prior on local neighbor relationships. A $k$NN-like algorithm is needed to find neighbors from the pairwise distance matrix with additional time cost.

The affinity matrix obtained by the binary metric is often relatively sparse since only point pairs in the same leaf node are assumed to be similar. Generally speaking, the leaf node should not be too small to account for the affinity of the dataset: randomized trees should provide sufficient similar candidate points in leaf nodes.

### 3.2 Continuous forest-based metric

Aside from the binary affinity, we propose a continuous forest-based metric based on the common path $\mathbb{P}_{ij}$ of two instances $t_i$ and $t_j$ as they traverse from the root to leaves $\ell(t_i)$ and $\ell(t_j)$. The distance $d_{\mathrm{cp}}(t_i, t_j)$ is computed by the common path as follows:

$$d_{\mathrm{cp}}(t_i, t_j) = \frac{\nu_{ij} - |\mathbb{P}_{ij}|_{\mathrm{o}}}{\nu_{ij}} \tag{3}$$

where $\nu_{ij} = \max(\nu_i, \nu_j)$ is the maximum depth of $\ell(t_i)$ and $\ell(t_j)$, and $|\cdot|_{\mathrm{o}}$ is the cardinality of a set. If two instances reach the same leaf node, the distance is zero. Otherwise, the distance is set to 1 when the two instances lack a common path. The binary affinity definition is a special case of Eq. (3) by setting the common path to null for instances not in the same leaf. However, there is no guarantee that the decision tree is balanced for an arbitrary dataset. In this case, similarity is defined based on the cardinality of the data stored in the smallest shared parent (SSP) node $T_{p_{ij}}$ of $\ell(t_i)$ and $\ell(t_j)$.

$$d_{\mathrm{sp}}(t_i, t_j) = \frac{|T_{p_{ij}}|_{\mathrm{o}} - \zeta_{ij}}{|T_r|_{\mathrm{o}} - \zeta_{ij}} \tag{4}$$

where $\zeta_{ij} = \min(|\ell(t_i)|_{\mathrm{o}}, |\ell(t_j)|_{\mathrm{o}})$ is the minimum leaf size of $\ell(t_i)$ and $\ell(t_j)$. When $t_i$ and $t_j$ go into the same leaf node, the SSP node $T_{p_{ij}}$ is the leaf itself, and distance $d_{\mathrm{sp}}$ is zero. On the other hand, when the shared parent node is at the highest level, i.e., the root node $T_r$, $d_{\mathrm{sp}}$ is set to 1. When the leaf size $n_l$ is selected as the termination criterion of the tree growth, the above SSP-based metric can be simplified to $d_{\mathrm{sp}}(t_i, t_j) = \vartheta(|T_{p_{ij}}|_{\mathrm{o}} - n_l)$, where the normalization constant $\vartheta = (|T_r|_{\mathrm{o}} - n_l)^{-1}$. For an unbalanced data distribution, the distance between two instances in a small cluster is shorter than in a large cluster, using the definition in Eq. (4): two instances are likely to be far apart in the large cluster. Compared to the adaptive forest-based metric in Ref. [25], here the cardinality of the SSP node is used to determine affinity without weight computation in the shared traversal path. The combined forest-based metric $d_{\mathrm{f}}$ is defined as a linear combination of the common path-based $d_{\mathrm{cp}}$ and the SSP-based $d_{\mathrm{sp}}$.

$$d_{\mathrm{f}} = w_{\mathrm{cp}} d_{\mathrm{cp}} + w_{\mathrm{sp}} d_{\mathrm{sp}} \tag{5}$$

where the constant weight $w_{\mathrm{cp}} + w_{\mathrm{sp}} = 1$. The entry in the affinity matrix $\mathcal{A}$ is defined as $\mathcal{A}_{ij} = 1 - d_f(t_i, t_j)$.

**Proposition 1.** *The functions defined in Eqs. (3)–(5) are non-negative metrics with following properties:*
- *Identity:* $d(t_i, t_i) = 0$.
- *Positivity:* $d(t_i, t_j) \geqslant 0$.
- *Symmetry:* $d(t_i, t_j) = d(t_j, t_i)$.
- *Triangle inequality:* $d(t_i, t_k) \leqslant d(t_i, t_j) + d(t_j, t_k)$.

The proof of Proposition 1 is given in Appendix B. The above binary, the common-path-based, the SSP-based, and the combined distance metrics are applied to a set of toy data in Figs. 2 and 3. The difference $e_{\mathcal{A}}$ between the affinity matrices $\mathcal{A}$ computed by the clustering forest-based metrics and $\mathcal{A}_{L_2}$ by the $L_2$ norm and the $k$NN is shown in Fig. 4.

$$e_{\mathcal{A}} = \frac{\|\mathcal{A} \oplus \mathcal{A}_{L_2}\|_{\mathrm{F}}^2}{n_{\mathcal{A}}} \tag{6}$$

where $\oplus$ is the *xor* operator of matrix entries. $n_{\mathcal{A}}$ is the size of $\mathcal{A}$. $\|\cdot\|_{\mathrm{F}}$ is the Frobenius norm. The combined random-forest-based metric achieves lower $e_{\mathcal{A}}$ than the binary, the common-path-based, and the SSP-based metrics. All metrics display a reduced difference $e_{\mathcal{A}}$ when enlarging the forest size. The Dice similarity metric [40] $e_I$ is used to compare the $k$ nearest neighbors obtained by the proposed metrics

**Fig. 2** Toy datasets: (a) *punctured sphere*, (b) *3D clusters*, (c) *twin peaks*, and (d) *corner*.

**Fig. 3** Affinity matrices obtained by the proposed forest-based metric and the $L_2$-norm followed by $k$NN on (a) *corner* and (b) *3D clusters* datasets.

with those from the $L_2$ norm in Fig. 5. The nearest neighbors obtained by the combined random-forest-based metric are more consistent with the $L_2$ metric than other metrics. We observe that consistency increases with increasing forest size. Moreover, on enlarging the forest size, the performance of the binary random-forest-based metric approaches that of the combined metric (see Figs. 4(a) and 5(a)), because a large number of randomized decision trees tend to provide sufficient neighboring candidates.

The look-up of feature values and comparison with thresholds when traversing trees are very fast and take negligible time. Although the cost of pairwise distances for small subsets or sampled point pairs is much lower than dense pairwise distance computation, any $k$NN-graph-based method is time-consuming for a high-dimensional dataset. The proposed forest traversal and leaf assignments have linear complexity with respect to the data size. More importantly, the time complexity of our method is independent of dimensionality, which is desirable for high-dimensional data. In the extreme case of a forest-based metric, the binary metric, there are no multiplication operations in the affinity estimation. Since the instances in the same leaf node are assumed to be similar, the complexity depends on the number of the leaf nodes, and there is no pairwise distance computation by the binary forest-based metric. For the continuous metrics, such as $d_{\mathrm{sp}}$, there are just normalization operations in the affinity estimation.

## 4 Pseudo leaf splitting

It is efficient to acquire the pairwise affinity matrix between datasets by the random-forest-based metric. However, there is no regularization for point-wise correspondence because the random forest is built upon independent feature descriptors without considering the relationship. For instance, when establishing correspondence $C$ between datasets $X$ and $Y$, the forest-based metric can be used to produce a candidate matching pair $\{(x_i, y_i) \in C | x_i \in X, y_i \in Y\}$. The above correspondence does not guarantee relationship preservation, i.e., $g(x_i, x_j) \propto g(y_i, y_j)$

**Fig. 4** Affinity matrix difference $e_{\mathcal{A}}$ for the combined forest-based metric (Fuse), the binary metric (Bin), the common-path-based metric (Path), and the SSP-based metric for four toy datasets: (a) *corner*, (b) *punctured sphere*, (c) *twin peaks*, and (d) *3D clusters*.

**Fig. 5** Dice similarity $e_I$ of nearest-neighbors obtained by the combined forest-based metric (Fuse), the binary metric (Bin), the common-path-based metric (Path), and the SSP-based metrics for four toy datasets: (a) *corner*, (b) *punctured sphere*, (c) *twin peaks*, and (d) *3D clusters*.

when $(x_i, y_i) \in C$ and $(x_j, y_j) \in C$. $g$ is some function to measure the relationship, e.g., the geodesic distance on a 3D mesh surface. This work introduces PLS to handle the lack of affinity regularization in the forest-based metric.

To begin with, the leaf node $\ell^*$ with the largest span is located as the starting leaf, and we set

$$\ell^* = \underset{x_i, x_j \in \ell}{\operatorname{argmax}}\, g(x_i, x_j) \tag{7}$$

The span of starting node $\ell^*$ is denoted $\eta^* = \max\limits_{x_i, x_j \in \ell^*} g(x_i, x_j)$. Generally speaking, the leaves of extreme points can be identified in this way, e.g., the leaf node of the toe in a 3D human mesh dataset. A Gaussian mixture model (GMM) is used to fit the point distribution in the leaf node. For simplicity, the dominant mode acquired by the mean shift method [41] is used to represent the leaf. Let $\mu_\ell^*$ denote the center of the dominant mode in $\ell^*$. Point $x^* \in \ell^*$ is selected as the seed satisfying

$$x^* = J(X) = \arg\min_{x \in \ell^*} \|x - \mu_\ell^*\| \tag{8}$$

$J(X)$ returns the seed point of dataset $X$. The point set belonging to $X$ and $\ell^*$ is split according to the seed selection. In our system, the seed point is assigned to the left leaflet. The binary test for leaf splitting is defined as

$$\varphi^*(x) = \begin{cases} 1, & \text{if } g(x, x^*) < 0.5\eta^* \\ 0, & \text{otherwise} \end{cases} \tag{9}$$

Given the starting leaf node and the seed selection, the leaf splitting is propagated to other leaves. The unprocessed leaves are sorted by distance to the seed point $x^* \in \ell^*$ and propagation begins from the nearest leaf node. Let $\ell_k$ be the current leaf node. For point $x \in \ell_k$, the binary test for leaf splitting of dataset $X$ is defined as

$$\varphi_k(x) = \begin{cases} 1, & \text{if } g(x, x^*) \leqslant 0.5(\eta_{k1} + \eta_{k2}) \\ 0, & \text{otherwise} \end{cases} \tag{10}$$

where $\eta_{k1} = \min\limits_{x \in \ell_k} g(x, x^*)$, and $\eta_{k2} = \max\limits_{x \in \ell_k} g(x, x^*)$. Only leaf nodes with ambiguous correspondence need to be split, which can be determined simply by checking the span of the leaf node. When the span is greater than the predefined threshold, set to 10% of the largest span of dataset $X$ in our experiments, the leaf nodes are split. The pseudo-leaf-splitting process is given in Algorithm 1.

PLS is a general technique to regularize the pairwise affinity obtained from a forest. Here the function $g$ is used to measure the point-wise relationship between points inside a dataset, where the leaflet splitting tests are set according to the span of the dataset. There are no requirements that two sets share the same span when using the forest-based metric and PLS regularization to establish point-wise correspondence. The proposed scheme can handle non-isometrically deformed datasets by using the data-dependent binary tests in Eqs. (9) and (10).

**Algorithm 1** Pseudo leaf splitting

**Input:** Random forest $R$, dataset $X$.
**Output:** Pseudo leaf splitting.
**for** Each tree in $R$ **do**
  Locate starting leaf $\ell^*$ with the largest span (Eq. (7));
  Compute the centroid of the dominant mode in $\ell^*$;
  Get a seed point $x^* \in \ell^*$ (Eq. (8));
  Split leaf node $\ell^*$ using Eq. (9);
  Sort unprocessed leaves by distance to $x^*$;
  **for** Each inconsistent leaf node **do**
    Perform leaf splitting using Eq. (10);
  **end for**
**end for**

It is computationally complex to find consistent correspondences in a shape corpus. Existing techniques do so by minimizing overall distortion using dynamic programming [36], positive semi-definite matrix decomposition [34], and functional map networks [35]. Additional refinement is required for consistent correspondence when given an initial pairwise mapping. The gap between the point-wise correspondence of shapes and the consistency refinement can be avoided by taking into account point distribution in the shape corpus. Unlike the example-based classification forest for shape correspondence [9], there is no need for labeled training data using the proposed forest-based metric.

The correspondence function between surface meshes $X^p$ and $X^q$ is denoted $\tau_{pq}(x_i^p) = x_j^q$, where affinity $\mathcal{A}_{ij}^{pq} = \max_{x_{j*}^q \in X^q} \mathcal{A}_{ij*}^{pq}$. When given a group of surface meshes, point-wise correspondence using PLS is consistent and satisfies cycle constraints: when $\tau_{pq}(x_i^p) = x_j^q$ and $\tau_{qr}(x_j^q) = x_k^r$, $\tau_{pr}(x_i^p) = x_k^r$. It can be ascribed to the seed selection based on the Gaussian fitting of the dominant mode in $\ell^*$. The mapping between starting seed points of $X_p$ and $X_q$ is $\tau_{pq}(x^{p*}) = J_q J_p^{-1}(x^{p*}) = x^{q*}$. It is obvious that correspondence of seed points satisfies cycle constraints, where $\tau_{pr}(x^{p*}) = J_r J_q^{-1} J_q J_p^{-1} = J_r J_p^{-1} = x^{r*}$. Taking into account the similarity propagation nature of PLS, the point-wise correspondence satisfies the cycle constraints.

## 5 Experiments

### 5.1 Datasets and metric

The proposed method is applied to affinity estimation of various datasets, including KinectVS [4], OULUVS [42], and OuluVS2 [43]. KinectVS consists of twenty subjects uttering twenty phrases six times [4]. Color and depth video data were obtained by *Kinect* with a resolution of $640 \times 480$. The OULUVS dataset [42] consists of color videos of twenty subjects uttering ten phrases five times with a resolution of $720 \times 576$. OuluVS2 [43] consists of color videos of 53 subjects uttering ten phrases three times with a resolution of $1920 \times 1080$.

The AAM algorithm [44] is used to extract 35 patch trajectories around the lips and jaw following Ref. [4], where the shape and texture features of patches are concatenated to represent the trajectories. In our experiments, the affinity matrix obtained by the forest-based metric is sorted, and $r$ nearest neighbors are viewed as matching candidates of probe trajectories. $r$ is set to 1 (Top-1), 5 (Top-5), and 10 (Top-10) in the affinity evaluation. If the trajectory with the same label as the probe occurs in the candidate set, there is a hit. The trajectory labeling accuracy is computed as $n_{\text{hit}}/n_{\text{probe}}$, where $n_{\text{hit}}$ and $n_{\text{probe}}$ denote the numbers of hits and probe trajectories respectively.

We also evaluate the proposed method on 3D shape corpora, including TOSCA [45], Scape [46], SHREC07-NonSym [34, 45], and Faust datasets [47]. The wave kernel signature (WKS) [48] and normalized geodesic distance vector are used as 3D point feature descriptors. The geodesic distance vector of point $x$ is composed of the geodesic distance between $x$ and all other points on the surface meshes, computed by the fast marching algorithm. The correspondence accuracy of 3D surface meshes $X$ and $Y$ is defined as

$$e_{XY} = \frac{1}{n_X} \sum_{i=1}^{n_X} g(\tau(x_i), \tau'(x_i)) \tag{11}$$

where $\tau$ and $\tau'$ are the estimated and ground truth point-wise mapping functions, $n_X$ is the number of points in $X$, and $g$ is the geodesic distance function. The percentages of correct matches with a set of geodesic errors, including 0.02, 0.05, 0.10, and 0.16, are reported in our experiments.

### 5.2 Affinity estimation

The proposed method is applied to affinity estimation on the facial trajectories and 3D points. We compare the proposed criteria with the classical Gini index [25], the determinant of the covariance matrix [20], and the variance of feature differences [23] on the facial trajectories (Figs. 6(a)–6(c)) and 3D shape datasets (Figs. 6(e)–6(g)). The node splitting criterion based on the determinant of the covariance matrix [20] fails for all datasets due to rank deficiency of the covariance matrices. The forests built by the Gini index of the dummy set [19, 24, 25] depend on the construction of synthetic data, being limited to locate the data clusters effectively. The node splitting criterion tries to find a feature pair to produce the largest variance of feature difference [23], which does not model the data distribution of child nodes. On the other hand, our splitting criteria handle the data distribution and produce the best results with the Fuse metric. The numbers of trees are set to 17 and 50 for the visual utterance datasets and 3D shape datasets respectively.

Comparisons of binary (Bin), common-path (Path), SSP, and combined distance metrics (Fuse) on the facial trajectories and 3D points are shown in Figs. 6(a)–6(c) and Figs. 6(e)–6(g). The Fuse metric shows better performance than the binary one, and produces an improvement relative to the Path and the SSP-based metrics. For two pairs with common paths of the same length, the one with the smaller SSP is more similar than the other. Both the Path and SSP metrics contribute to affinity estimation based on tree traversal in forests.

Figures 6(d) and 6(h) show the labeling accuracies of the facial trajectories for KinectVS, OuluVS, and OuluVS2, as well as the 3D point matching accuracies on TOSCA, Scape, and Shrec-NonSym datasets with and without PLS regularization. The labeling results with PLS regularization are better than those without for all datasets. Because the shape feature defined as the difference of patch positions in adjacent frames possesses motion information, the symmetric facial trajectories on the left and right half faces are less likely to be confused. Thus, improvements using PLS regularization are limited for the facial trajectory datasets compared to those for the 3D shape datasets.

The facial tracker is designed for frontal faces, and tracking performance deteriorates when given

**Fig. 6** Labeling accuracies for the combined random-forest-based metric (Fuse), the binary (Bin), common-path-based (Path), and SSP-based metrics, random forests with node splitting criterion using the determinant of the covariance matrix [20], the variance of feature differences [23], and the Gini index [25] on (a) KinectVS, (b) OuluVS, (c) OuluVS2, (e) TOSCA, (f) Scape, and (g) Shrec-NonSym datasets. The Top-5 and g0.02 accuracies with and without PLS on facial trajectories and 3D points are shown in (d) and (h) respectively.

profile facial images in the OuluVS2 phrase dataset [43]. Figure 7 shows the effects of facial landmark tracking on affinity estimation for trajectories. The less accurate facial landmark tracking in the profile views makes it harder to locate the correct facial trajectories. The facial trajectory labeling accuracy of the frontal view is better than for the profile views in the Top-1, Top-5, and Top-10 experiments.

### 5.3 Dense correspondence between shapes

An unsupervised random forest-based metric with PLS regularization is employed to estimate the point distribution (see Fig. 8). A comparison of the pairwise correspondence found by the proposed method and functional maps (FM) [6], blended intrinsic maps (BIM) [7], a coarse-to-fine combinatorial method [8], and a classification random forest (CRF) [9], are shown in Table 1. Like Ref. [9], we only conduct experiments on the classes with more than six objects to ensure sufficient training data for the forest. Here all shapes except the query are used to train the forest. Our method can achieve more than 96% correct matching within 0.05 geodesic error. In the experiments, the WKS and the geodesic distance vectors are used as the point descriptor.

**Fig. 8** Pairwise shape correspondence between (a) reference and target shapes (b) without and (c) with the PLS regularization. (d) and (e) are the affinity matrix for the cat and the dog without PLS.

**Fig. 7** Labeling accuracies for facial trajectories on OuluVS2 of different camera views including 0°, 30°, and 60°.

Table 1 gives the correspondence accuracy based on WKS ($RF_{wks}$), the geodesic distance vector ($RF_{geo}$), and feature fusion ($RF_{fusion}$). In our experiment, the accuracy of the dense correspondence given by $RF_{fusion}$ outperforms those from $RF_{wks}$ and $RF_{geo}$. The fusion of the local shape descriptor WKS and the contextual geodesic vector facilitates searching for the optimal node splitting.

**Table 1** Comparison of pairwise correspondences by the proposed method with and without PLS regularization, and combinatorial [8], FM [6], BIM [7], and CRF [9] methods on the TOSCA dataset

| Method | Correspondence (%) | | | |
|---|---|---|---|---|
| | g0.02 | g0.05 | g0.10 | g0.16 |
| Combinatorial [8] | 24.8 | 56.0 | 80.8 | 90.5 |
| BIM [7] | 44.3 | 84.6 | 95.7 | 97.7 |
| FM [6] | 66.5 | 86.8 | 94.0 | 96.7 |
| CRF [9] | 65.6 | 94.5 | 99.1 | 99.2 |
| $RF_{geo}$ | 21.9 | 46.3 | 71.7 | 84.2 |
| $RF_{wks}$ | 44.8 | 84.1 | 93.1 | 96.2 |
| $RF_{fusion}$ | **67.3** | **96.5** | **99.4** | **100** |
| w/o PLS | 35.6 | 63.3 | 72.5 | 79.8 |

Point-wise matching based on forests with different numbers of trees is shown in Fig. 9(a). The forest size is larger than that for the supervised CRF [9]. A relatively large number of randomized decision trees is needed to estimate the correspondence in an unsupervised manner. The more the training data, the more accurately the correspondence can be obtained (see Fig. 9(b)).

We have applied the proposed method to a motion dataset [49], where the first 10% shapes are used to train the forest. There is no requirement that the training and testing shapes are from the same kind of motions. Our method can achieve more than 95% correct matches within 0.05 geodesic error, as shown in Fig. 9(c).

The proposed method is compared with convex-optimization-based nonrigid registration (CO) [51] and a CNN classifier-based method [52] on the Faust database [47]. Following Refs. [51, 52], correspondence is computed between pairs of meshes from the same subject (intra-subject) or different subjects (inter-subject). Aside from the testing pairs, all other meshes are used to build the random forest. Our method outperforms CO and the CNN-based method in average error, average error of the worst pair, and 10-cm recall: see Fig. 10. CNN followed by nonrigid registration (CNN-S) produced the best results. However, CNN and CNN-S were built upon 2D depth maps, where partial scans and additional registration operations were required.

Figure 11 and Table 2 show a comparison with deep learning-based shape correspondence models, including 3D-coded [38], FMNet [37], and ADD3 [5]

**Fig. 9** (a) Correspondence errors with different forest sizes for the TOSCA dataset. (b) Average geodesic errors corresponding to various sizes of training sets for the proposed method ($RF_{fusion}$) and a classification random forest (CRF) [9]. (c) Comparison of pairwise correspondence errors with different feature channels on human motion data [49]. (d) Comparison of consistent correspondence errors on the SHREC-NonSym dataset by the proposed method, and FMN [35], SDP [34], CFM [50], and OBF [36] methods.

**Fig. 10** Comparison in terms of (a) average error, (b) average error on the worst pair, (c) 10 cm recall for the proposed method, CO [51], CNN [52], and CNN-S [52] on the Faust dataset.

**Fig. 11** Comparison with deep learning-based methods. (a) Reference. (b, c) Proposed method without and with the PLS regularization respectively. (d) 3D-coded [38]. (e) FMNet [37].

**Table 2** Comparison of the proposed method to deep learning-based shape correspondence methods on the Scape dataset

| Method | 3D-coded [38] | FMNet [37] | ADD3 [5] | Ours |
|---|---|---|---|---|
| g0.02 | 0.48 | **0.78** | 0.27 | 0.65 |

on the Scape dataset The proposed forest-based metric with PLS regularization outperforms the supervised and unsupervised deep learning-based models with a matching accuracy of 0.65 vs. 0.48 (3D-coded) and 0.27 (ADD3) at g0.02. The supervised FMNet has the best performance, which is learned from prior ground truth correspondence and the mapping in both the spatial and spectral domains. On the other hand, the proposed approach only requires unsupervised forest-based metric learning for point-wise affinity.

## 5.4 Consistent correspondence in shape corpus

Aside from pairwise shape correspondence, the proposed method is also compared with existing consistent correspondence methods, including positive semi-definite matrix decomposition (SDP) [34], an optimization-based framework (OBF) for distortion minimization [36], a functional map network (FMN) [35], and consistent functional maps (CFM) [50] on the SHREC-NonSym dataset: see Table 3 and Fig. 9(d). The proposed method takes advantage of the point distribution modeling by the clustering forest and the PLS regularization scheme, outperforming the compared methods with correspondence accuracies of 44.2% (g0.02) on the Shrec-NonSym dataset.

Table 4 gives the correspondence by the proposed method, SDP [34], OBF [36], and fuzzy correspondence (FC) [53] methods on the TOSCA and Scape datasets. The proposed method outperforms SDP [34] and OBF [36] by significant margins in local matching with 0.02 geodesic errors: the proposed method has an edge in matching specificity. At 0.16

**Table 3** Comparison of consistent correspondence by the proposed $RF_{fusion}$ method with and without PLS regularization, and FMN [35], SDP [34], CFM [50], and OBF [36] methods, on the Shrec-NonSym dataset

| Method | Correspondence (%) | | | |
|---|---|---|---|---|
| | g0.02 | g0.05 | g0.10 | g0.16 |
| FMN [35] | 42.7 | 70.9 | 89.8 | 95.8 |
| SDP [34] | 16.9 | 45.6 | 71.7 | 85.7 |
| CFM [50] | 12.8 | 36.0 | 68.3 | 84.5 |
| OBF [36] | 19.5 | 47.8 | 70.2 | 79.0 |
| $RF_{fusion}$ | **44.2** | **74.3** | **90.9** | **97.1** |
| w/o PLS | 15.8 | 30.6 | 58.9 | 69.4 |

**Table 4** Comparison of matching at 0.02 (g0.02) and 0.16 (g0.16) geodesic errors by the proposed $RF_{fusion}$ method with and without the PLS regularization, and SDP [34], OBF [36], and FC (♯) [53] methods on the TOSCA and Scape datasets

| | Error | SDP [34] | OBF [36] | $RF_{fusion}$ | w/o PLS |
|---|---|---|---|---|---|
| TOSCA | g0.16 | **100** | 97.6 | **100** | 79.8 |
| | g0.02 | 34.1 | 37.5 | **60.2** | 35.6 |
| Scape | g0.16 | **100** | **100** | **100** | 77.3 |
| | g0.02 | 41.2 | 48.6♯ [53] | **65.3** | 34.8 |

geodesic errors, the proposed method can realize full matching as SDP [34] and OBF [36] methods.

As shown in Tables 1, 3, and 4, the proposed forest-based metric with PLS regularization refines the forest-based metric and produces an improvement by a large margin for both pairwise and consistent correspondence in the shape corpus.

## 5.5 Phrase recognition

Phrase recognition accuracies for the proposed method ($RF_{fusion}$) on the depth and color videos are given in Fig. 12. The accuracy for subject-independent (SI) experiments is lower than for subject-dependent (SD) experiments. The performance variations in the SD and SI experiments can be ascribed to personal speaking characteristics and person-specific texture differences regarding the moustache and lip shapes. The SI experiments on the frontal phrase set of OuluVS2 provide an average accuracy of 84.8%, comparable to the state-of-the-art methods [54, 55] (see Fig. 13). In the OuluVS2 dataset, the video data for Subject 29 turned out to be unusable since his mouth was not seen most of the time, so Subject 29 was not used in the test data.

Table 5 reports the phrase recognition accuracies on the frontal phrase set of the OuluVS2 dataset in SI experiments. The proposed model is compared to deep CNN-based lipreading models with the long

**Fig. 12** Phrase recognition accuracies for each subject in (a) KinectVS and (b) OuluVS datasets by the $\mathrm{RF}_{\mathrm{fusion}}$ in the subject-dependent (SD) and independent (SI) experiments.

**Fig. 13** Phrase recognition accuracies for each subject from the OuluVS2 phrase dataset.

**Table 5** Phrase recognition accuracies on the OuluVS2 dataset

| Method | Zhou [54] | Lee [55] | Chung [56] | Chung [57] | Ours |
|---|---|---|---|---|---|
| Accuracy (%) | 73.5 | 81.1 | 93.2 | **94.1** | 84.8 |

short-term memory architecture [55] and parallel branches [56]. A large-scale dataset was used to learn the network parameters [57]. The proposed model achieves an average accuracy of 84.8%, comparable to a latent variable model [54] and LSTM [55]. The system based on deep neural networks produces a large margin improvement [56, 57]; many parameters need to be learned from annotated training data.

Figure 14 gives phrase recognition accuracies for each subject in the color videos ($\mathrm{RF}_{\mathrm{color}}$) with a patch size of $15 \times 15$ and the depth videos ($\mathrm{RF}_{\mathrm{depth}}$) with a patch size of $7 \times 7$, for the KinectVS dataset. We set the patch sizes following Ref. [4]; the patch size for depth videos is smaller given the relatively low signal-to-noise ratio of the depth video.

**Fig. 14** Phrase recognition accuracies for each subject in the color videos ($\mathrm{RF}_{\mathrm{color}}$) with a patch size of $15 \times 15$, and the depth videos ($\mathrm{RF}_{\mathrm{depth}}$) with a patch size of $7 \times 7$, for the KinectVS dataset.

### 5.6 Comparison with forest-based correspondence

The proposed method utilizes a multivariate Gaussian distribution and clustering forest-based metrics for affinity estimation and correspondence. We estimate supervoxel correspondence on bony tissues of the craniofacial CBCTs, which are divided into two parts, the mandible and the maxilla. The dataset consists of 150 clinically obtained cone beam CTs (CBCTs) [30], each decomposed into 5000 supervoxels. We compare with recent work on forest-based metrics, including OCF [29], MMRF [28], SC forest [30], and the classification forest (CLA) [58], on supervoxel-wise correspondence; see Table 6. In experiments, we estimate the supervoxel-wise correspondence on bony tissues of the craniofacial CBCTs. The proposed approach extends the binary forest-based metric to a continuous one, and achieves a Dice similarity coefficient (DSC) of 0.93 on the maxilla, outperforming MMRF (0.88), SC (0.89), and CLA (0.81). MMRF and SC show better performance for the mandible with a relatively small number of supervoxels than ours, though additional classification criteria and tree pruning are required. Here OCF achieves the best performance with DSC of 0.93 and 0.95 on the mandible and the maxilla respectively. However, OCF requires additional dominant principal component estimation and regression [29]. The proposed approach does not incur additional computational cost to forest construction.

**Table 6** Comparison on supervoxel-wise correspondence by forest-based methods

| | MMRF [28] | SC [30] | OCF [29] | CLA [58] | Ours |
|---|---|---|---|---|---|
| Mandible | 0.91 | 0.92 | **0.93** | 0.88 | 0.88 |
| Maxilla | 0.88 | 0.89 | **0.95** | 0.81 | 0.93 |

## 6 Conclusions

We have presented unsupervised random-forest-based metrics for affinity estimation for large and high-dimensional data, taking advantage of both the common traversal path and the smallest shared parent node. The proposed forest-based metric combined with PLS can account for spatial relationships to determine consistent correspondences. The proposed PLS scheme regularizes the forest-based metric and avoids the gap between point-wise correspondence and additional consistency refinements inside a shape corpus. The proposed method has been applied to phrase recognition using color and depth videos, as well as point-wise correspondence of 3D shapes, demonstrating the effectiveness of the proposed method compared to the state-of-the-art.

In future, we will further explore clustering random forest methods for affinity estimation. The additional PLS is utilized in the current system to account for global spatial and structural relationships. This approach can regularize the forest-based metric but relies on a heuristic seed selection and propagation process to optimize the node splitting parameters and generate the forest. We will further study optimization of unsupervised clustering forests for consistent and point-wise correspondence.

## Appendix A Decremental covariance matrix evaluation

Since the covariance matrices need to be evaluated repeatedly when given randomly selected parameters, it is time consuming to evaluate the covariance matrix $\sigma$ from scratch for the optimal splitting parameters when building the forest. Let $\rho$ be the data dimensionality. The time complexity of covariance matrix construction is $O(\kappa \min(m_{T_l}^2 \rho, m_{T_l}\rho^2) + \kappa \min(m_{T_r}^2 \rho, m_{T_r}\rho^2))$ for $\kappa$ randomly selected parameters. The complexity of trace evaluation is $O(\kappa m_{T_l}\rho + \kappa m_{T_r}\rho)$. The decremental evaluation technique for covariance matrices is presented using the fact that the data in each node are a subset of that of the root node.

Let $\sigma_p$, $\sigma_l$, $\sigma_r$ denote the covariance matrices of the parent and two child nodes respectively. The $ij$-th entry of $\sigma_p$ is defined as $\sigma_{p_{ij}} = \boldsymbol{E}((t_i-\mu_p)(t_j-\mu_p)')$. Without loss of generality, here the left child node is assumed to be larger than the right one. To begin with, the covariance matrix of the smaller child node, i.e., the right one, is computed. The entry of $\sigma_r$ is defined as $\sigma_{r_{ij}} = \boldsymbol{E}((t_i-\mu_r)(t_j-\mu_r)')$. For a point pair $(t_i, t_j)$ belonging to both the parent and the right child nodes, the differences of corresponding entries in $\sigma_p$ and $\sigma_r$ are computed as follows.

$$\widetilde{\sigma}_{p_{ij}} - \widetilde{\sigma}_{r_{ij}} = -(t_i+t_j)(\mu_p-\mu_r)' + \|\mu_p\|^2 - \|\mu_r\|^2 \quad (12)$$

where $\widetilde{\sigma}_{p_{ij}} = \sigma_{p_{ij}}(m_{T_p}-1)$ and $\widetilde{\sigma}_{r_{ij}} = \sigma_{r_{ij}}(m_{T_r}-1)$. Let $\sigma_r^*$ denote the sub-matrix of $\sigma_p$ with columns and rows corresponding to points in the right child node.

The trace of the covariance matrix $\sigma_r$ of the right child node is derived as

$$\mathrm{tr}(\sigma_r) = \frac{\mathrm{tr}(\sigma_r^*)(m_{T_p}-1) + 2\sum_{i=1}^{m_{T_r}} t_i o_r' - m_{T_r}\mathfrak{o}_r}{m_{T_r}-1} \quad (13)$$

where $o_r$ is the displacement vector from the centroid of the right child node to the parent, and $o_r = \mu_p - \mu_r$. The right child-related constant $\mathfrak{o}_r = \|\mu_p\|^2 - \|\mu_r\|^2$. Given $\mathrm{tr}(\sigma_r)$, the trace of $\sigma_l$ is computed as follows:

$$\mathrm{tr}(\sigma_l) = \frac{\mathrm{tr}(\sigma_p)(m_{T_p}-1) - \mathrm{tr}(\sigma_r)(m_{T_r}-1) + \mathfrak{o}_l}{m_{T_l}-1} \quad (14)$$

where $\mathfrak{o}_l = m_{T_p}\|\mu_p\|^2 - m_{T_r}\|\mu_r\|^2 - m_{T_l}\|\mu_l\|^2$.

Given the randomly selected splitting parameters, the centroids $\mu_l$ and $\mu_r$ of the left and right child nodes, as well as the norms $\|\mu_l\|$ and $\|\mu_r\|$ are computed. Next, the trace of the covariance matrix of the smaller child node is computed based on the submatrix extracted from the parent node as in Eq. (13). The trace of the covariance matrix of the other child node is computed given $\mathrm{tr}(\sigma_p)$ and $\mathrm{tr}(\sigma_r)$ as in Eq. (14). Since just the traces of the covariance matrices are needed to estimate the information gain in our system, the complexity of the covariance matrix evaluation is reduced from $O(m_{T_l}\rho + m_{T_r}\rho)$ to $O(\rho)$. Given $\kappa$ randomly selected parameters, the trace evaluation complexity is reduced to $O(\kappa\rho)$.

## Appendix B Proof of Proposition 1

We prove the functions defined in Eqs. (3)–(5) are metrics as follows.

**Eq. (3)**

Let $t_i, t_j, t_k$ be three input instances and the corresponding leaf nodes be $\ell(t_i), \ell(t_j), \ell(t_k)$. The common paths are denoted $\mathbb{P}_{ij}, \mathbb{P}_{jk}$, and $\mathbb{P}_{ik}$.

*Identity*: $d_{\mathrm{cp}}(t_i, t_i) = (|\mathbb{P}_{ii}|_{\mathrm{o}} - |\mathbb{P}_{ii}|_{\mathrm{o}})/\nu_{ii} = 0$;

*Positivity*: Because $|\mathbb{P}_{ij}|_{\mathrm{o}} \leqslant \nu_i$ and $|\mathbb{P}_{ij}|_{\mathrm{o}} \leqslant \nu_j$, $|\mathbb{P}_{ij}|_{\mathrm{o}} \leqslant \nu_{ij}$. Thus, $d_{\mathrm{cp}}(t_i, t_j) = (\nu_{ij} - |\mathbb{P}_{ij}|_{\mathrm{o}})/\nu_{ij} \geqslant 0$;

*Symmetry*: $|\mathbb{P}_{ij}|_{\mathrm{o}} = |\mathbb{P}_{ji}|_{\mathrm{o}}$, so $d_{\mathrm{cp}}(t_i, t_j) = d_{\mathrm{cp}}(t_j, t_i)$;

*Triangle inequality*: Suppose that $\mathbb{P}_{ij}$ is the longest common path. Then $|\mathbb{P}_{ij}|_{\mathrm{o}} \geqslant |\mathbb{P}_{ik}|_{\mathrm{o}}$ and $|\mathbb{P}_{ij}|_{\mathrm{o}} \geqslant |\mathbb{P}_{jk}|_{\mathrm{o}}$. It follows that $|\mathbb{P}_{ik}|_{\mathrm{o}} = |\mathbb{P}_{jk}|_{\mathrm{o}}$ and $d_{\mathrm{cp}}(t_j, t_k) = d_{\mathrm{cp}}(t_k, t_i) \geqslant d_{\mathrm{cp}}(t_i, t_j)$. Thus,

$d_{\mathrm{cp}}(t_j,t_k) \leqslant d_{\mathrm{cp}}(t_i,t_j) + d_{\mathrm{cp}}(t_i,t_k)$, $d_{\mathrm{cp}}(t_i,t_k) \leqslant d_{\mathrm{cp}}(t_i,t_j) + d_{\mathrm{cp}}(t_j,t_k)$, and $d_{\mathrm{cp}}(t_i,t_j) \leqslant d_{\mathrm{cp}}(t_i,t_k) + d_{\mathrm{cp}}(t_j,t_k)$.

Similarly, when $\mathbb{P}_{jk}$ or $\mathbb{P}_{ik}$ is the longest common path, the triangle inequality property holds.

**Eq. (4)**

*Identity*: $d_{\mathrm{sp}}(t_i,t_i) = (|T_{p_{ii}}|_{\mathrm{o}} - |T_{p_{ii}}|_{\mathrm{o}})/(|T_r|_{\mathrm{o}} - |T_{p_{ii}}|_{\mathrm{o}}) = 0$;

*Positivity*: Because $|T_{p_{ij}}|_{\mathrm{o}} \geqslant \zeta_{ij}$ and $|T_r|_{\mathrm{o}} \geqslant \zeta_{ij}$, $d_{\mathrm{sp}}(t_i,t_j) \geqslant 0$;

*Symmetry*: $|T_{p_{ij}}|_{\mathrm{o}} = |T_{p_{ji}}|_{\mathrm{o}}$, so $d_{\mathrm{sp}}(t_i,t_j) = d_{\mathrm{sp}}(t_j,t_i)$;

*Triangle inequality*: Suppose that $T_{p_{ji}}$ is the smallest shared parent node. It follows that $|T_{p_{ik}}|_{\mathrm{o}} = |T_{p_{jk}}|_{\mathrm{o}}$ and $d_{\mathrm{sp}}(t_j,t_k) = d_{\mathrm{sp}}(t_k,t_i) \geqslant d_{\mathrm{sp}}(t_i,t_j)$. Thus, $d_{\mathrm{sp}}(t_j,t_k) \leqslant d_{\mathrm{sp}}(t_i,t_j)+d_{\mathrm{sp}}(t_i,t_k)$, $d_{\mathrm{sp}}(t_i,t_k) \leqslant d_{\mathrm{sp}}(t_i,t_j) + d_{\mathrm{sp}}(t_j,t_k)$, and $d_{\mathrm{sp}}(t_i,t_j) \leqslant d_{\mathrm{sp}}(t_i,t_k) + d_{\mathrm{sp}}(t_j,t_k)$.

Similarly, when $T_{p_{jk}}$ or $T_{p_{ik}}$ is the smallest shared parent node, the triangle inequality property holds.

## Eq. (5)

Since the function is a weighted combination of two metrics as defined in Eqs. (3) and (4), it is obvious that the function defined in Eq. (5) is a metric.

## Acknowledgements

This work was supported in part by the National Natural Science Foundation of China under Grant Nos. 61876008 and 82071172, Beijing Natural Science Foundation under Grant No. 7192227, and the Research Center of Engineering and Technology for Digital Dentistry, the Ministry of Health.
